# “Test me and treat me”—attitudes to vitamin D deficiency and supplementation: a qualitative study

**DOI:** 10.1136/bmjopen-2014-007401

**Published:** 2015-07-14

**Authors:** Siddharth Kotta, Dev Gadhvi, Niki Jakeways, Maryum Saeed, Ratna Sohanpal, Sally Hull, Olufunke Famakin, Adrian Martineau, Chris Griffiths

**Affiliations:** 1 Centre for Primary Care and Public Health, Asthma UK Centre for Applied Research, Blizard Institute, Queen Mary University of London, London, UK; 2 Department of Clinical Chemistry, Homerton University Hospital NHS Foundation Trust, Homerton Row, London, UK

**Keywords:** PRIMARY CARE, PUBLIC HEALTH, QUALITATIVE RESEARCH

## Abstract

**Objective:**

Lay interest in vitamin D and the potential benefits of supplementation is considerable, but little information exists concerning lay knowledge, beliefs and attitudes towards vitamin D to inform public health initiatives and professional guidance.

**Design:**

Qualitative focus group study.

**Participants:**

58 adults capturing diversity in disease status, gender, age and ethnicity.

**Setting:**

A large general practice in east London.

**Results:**

Many respondents lacked knowledge about vitamin D, including dietary sources and government recommendations. Most were positive about sun exposure, but confused by ambiguous health messages about risks and benefits of sunshine. Medicalised views of vitamin D were prominent, notably from those in favour of supplementation, who talked of “doses”, “side effects” and “regular testing.” Fortification of food with vitamin D was controversial, with opposing utilitarian (better overall for the majority) and libertarian (freedom to choose) views.

**Conclusions:**

Knowledge about vitamin D was limited. Clearer messages are needed about risks and benefits of sun exposure. Testing and supplementation by health professionals, while potentially useful in some high-risk groups, have contributed to a medicalised view of vitamin D. Health policy should address the public's need for clear information on sources and effects of vitamin D, including risks and benefits of sun exposure, and take account of divergent views on fortification. Professional guidance is needed on testing and supplementation to counter inappropriate medicalisation.

**Strengths and limitations of this study**

- We used qualitative methods to gather an in-depth understanding of people's knowledge and attitudes to aspects of vitamin D, including testing and supplementation.
- We gathered data from a wide range of people, covering people with and without illness, and from different ethnic backgrounds.
- As little is known about people's knowledge and attitudes to vitamin D, our data is important for people making health-policy recommendations about vitamin D.

## Introduction

**Figure 1:**
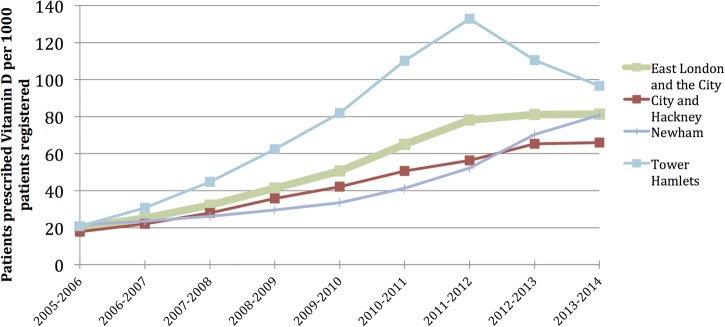
Numbers of patients prescribed vitamin D by general practices in the east London boroughs of Hackney, Tower Hamlets and Newham, and all three boroughs together (‘East London and the City’).

The last decade has seen an explosion of public interest in vitamin D. Use of vitamin D as a Google search term increased fivefold over the last decade.^1^ Vitamin D supplements and fortified foods are widely marketed as benefiting health. Widespread testing of vitamin D status and prescribing by health professionals has further fuelled public interest.^2^ One east London hospital laboratory processed a 10-fold increase in vitamin D test requests (largely from primary care) over a 5-year period from 2006 to 2010, reaching 44 500 per annum (personal communication, Timms P. Vitamin D testing at Homerton Hospital NHS Foundation Trust, 2014). Prescribing of vitamin D preparations has risen dramatically, with eight in every 100 east London patients receiving vitamin D (figure 1).^3^ In one east London borough (Tower Hamlets), numbers of patients prescribed vitamin D outstripped that for statins, aspirin, and proton pump inhibitors.^3^

Vitamin D deficiency is common and is associated with a range of illnesses beyond traditional diseases of bone (rickets and osteomalacia), including cancer, infectious disease and long-term conditions.^4–11^ Vitamin D may play key roles in regulatory systems, including host defence, immunity and repair.^4^ ^12^ However, considerable uncertainty surrounds the clinical significance of deficiency and the impact of supplementation. Clinical trials are beginning to clarify the effects of supplementation, with some consensus on the benefits on skeletal health^13^ ^14^ and the elderly,^15^ but otherwise largely inconsistent results.^13–18^ Deficiency and supplementation have moved from being subjects of scientific interest to public conversation. With respect to public health and policy, a number of questions arise. What does the public know about vitamin D? What are their views on testing, sun exposure, supplementation and food fortification? Do views vary between sections of society? Without an understanding of these issues, public health recommendations risk being poorly targeted and ineffective.

We, therefore, completed a focus group interview study to explore public knowledge of and attitudes to vitamin D. Our work was part of a National Institute for Health Research (NIHR)-funded programme of randomised trials examining the effects of supplementation in people with asthma, with chronic obstructive pulmonary disease (COPD), and in the elderly, and which took place in an area of ethnic diversity. We were, therefore, interested in learning the views of people from a range of ethnic groups, both healthy and with respiratory illness.

## Methods

### Sampling

Participants were recruited from a large general practice in east London. We used purposive sampling to ensure we gathered data from a range of ethnic groups, from people who were healthy or who had asthma or COPD, and a range of ages. We recruited three categories of patients, two of which reflected the groups eligible to join clinical trials we were conducting, while one-third comprised healthy adults under 65 years.

- Healthy adults under 65 years (2 groups)
- Adults over 65 years (2 groups)
- Adults with asthma or COPD (5 groups)

We identified potential participants by searching the practice computer system and invited them by letter. Ethnicity was identified using self-reported ethnicity as recorded by the practice. All focus groups occurred at the practice, except for the Bangladeshi group, which was carried out at a patient's home. All respondents were fluent in English.

Focus groups were facilitated by trained researchers who used two methods to stimulate discussion: in the first, they read out a series of statements about vitamin D (some deliberately incorrect), asking respondents to offer their views as to their veracity and in the second, they used a topic guide to ensure the subsequent discussion covered a full breadth of topics. We used an iterative process to influence further data collection, allowing emergent themes to be explored in subsequent groups. Focus group discussions were digitally recorded and fully transcribed.

### Data handling and analysis

Framework, a method widely used for applied or policy research, was used to carry out a thematic analysis.^14^ ^19^ Although the framework begins deductively with preset aims and objectives, there is an inductive ‘grounded’ reflection of the textual data. The five steps comprise familiarisation, developing a thematic framework, indexing, charting, mapping and interpretation.^14^ We used MAXQDA software to handle transcripts and Microsoft Excel for charting. Two transcripts were coded independently by three researchers (DG, NJ and SK). To improve inter-rater reliability, all three researchers cross-referenced their coding and from this a unified coding scheme was created.

## Results

Despite diversity in age, ethnicity and health, the knowledge and views of the nine groups showed more similarities than differences, and this is reflected in the text where we refer to specific groups only where there appeared to be important divergence. Participants had a median age of 58 years (range 25–85 years); just over two-thirds (40 of 58) were female; half (29) had either asthma or COPD and were recruited from a range of ethnic groups common in east London (table 1). Analysis generated three major categories with 18 subcategories. These were refined to produce three overarching themes (box 1).

Box 1 **Main themes arising from the data**

- Knowledge of and attitudes towards vitamin D
- Views about sunshine and sun exposure
- Attitudes towards testing and supplementation

**Table 1:** Participants

Group	Total	Male	Female	Average age (range)
Adults with asthma or COPD				
African	7	4	3	55.0 (34–66)
Bangladeshi	5	0	5	34.8 (29–39)
Black British	7	1	6	53.4 (39–63)
Indian or Pakistani	5	1	4	52.6 (41–65)
White British	5	2	3	55.4 (26–73)
Adults over 65 years				
Group 1	10	4	6	73.1 (65–85)
Group 2	11	1	10	69.9 (66–78)
Healthy adults under 65 years				
Group 1	4	2	2	35.0 (29–40)
Group 2	5	3	2	48.4 (25–60)
Total	58	18	40	57.8 (25–85)

COPD, chronic obstructive pulmonary disease.

## Knowledge and attitudes towards vitamin D

### Sources of information

The internet (typically “Google”), newspapers, magazines, pharmacists, doctors and leaflets/posters in surgery waiting rooms were common answers when participants were asked where they had read or heard about vitamin D, or where they would go to find out more. The internet was, however, described as confusing—“a nightmare”—by some because information was difficult to verify. Some used strategies to improve validity, for example, by using websites that looked “authoritative”, checking information from various websites, checking out only UK websites or searching websites with references. Advice from their doctor, pharmacist or nurse was more reliable because it could be “specific to an individual and to illnesses,” and so provided personalised advice. Most people from the over 65 years age groups had knowledge of vitamin D from their parents and public health campaigns during their youth, such as daily cod liver oil dosing in schools and public awareness of rickets in the 1950s (box 2).

Box 2 **Sources of information about vitamin D**

Difficulties with the internet:

- You have to check [the internet] because sometimes it is correct and sometimes it's not, and you find that…you start at the top, and by the time you've got to the bottom, you don't know if the information that you've seen before is correct and you think ohh, it just becomes a nightmare eventually, that's why I go straight to the pharmacist
  Black British, male
- I do a Google search and then, yeah, go for the more academic ones and see if I can make sense of them
  Elderly group, female

The need for information and advice to be tailored to each individual:

- I don't know. I think I'd want to talk to my doctor (murmurs of agreement) or kind of want to talk a nurse (more agreement) because you know there's general information but it's also very specific to individuals, (more agreement) around ill-…you know, things that have happened to you in the past and things that are happening to you now. But I think it's right to be sort of sceptical, so I would rather talk about an individual situation than just generally
  Elderly group, female

### Knowledge and beliefs

Knowledge in these respondents ranged from minimal—“All I know about vitamin D is the sunshine thing” or vague “helps keep you fit”, to erroneous “[vitamin D] comes from vegetables”—and the well-researched—“there's some indication it protects you against cancer…and coronary heart disease, but it…might [only] be an association.” Notably, respondents with limited knowledge of vitamin D predominated in all the groups interviewed. While almost all participants agreed vitamin D is important for healthy bones—“lack of vitamin D…starts to shrink [them] or something”—and that sunshine boosts vitamin D production, roughly half believed vegetables contained vitamin D. Participants from only two focus groups (1 over 65 years and 1 healthy) were clear that vegetables were not a good source of vitamin D. Oily fish and dairy products were commonly cited as good sources of vitamin D (although egg yolks are relatively poor).^4^ Almost all were unaware that sunscreen lotions block cutaneous vitamin D synthesis.

Respondents from the healthy and over 65 years age groups appeared better informed than those from other groups, citing beneficial effects of vitamin D that they had read in newspapers or on the internet. They knew of studies suggesting links to cardiovascular disease, Alzheimer's disease and the immune system. The over 65 years age groups displayed the greatest knowledge about dietary sources, associations with sunshine and wider awareness of vitamin D, perhaps reflecting their experience of public health campaigns during their youth, and because many had researched on this in their own time following prescriptions from their general practitioner (box 3).

Box 3 **Knowledge and beliefs**

Variable knowledge about vitamin D:

- Well, I believe from the sun really. I think chicken and orange or so
  African group
- I would say from vegetables. Fish and vegetables
  African group
- I just think so, I think so, maybe, something to do with eyes
  South Asian group
- I don't know enough to know either way. All I know about Vitamin D is the sunshine thing
  Healthy group
- I think people coming from hot climates, coming to live here in England and keeping the diet that they had at home, probably would mean that they perhaps would not be eating the oily fishes here. So they would be more at risk obviously to be lacking vitamin D
  Over 65 years age group
- When I was little, my mum used to give me this fish oil capsule and I was always keeping away!
  South Asian group
- There's some indication it protects you against cancer and protects you against coronary heart disease, but it's not highly decisive; it just looks like there might be an association
  Over 65 years age group
- If you look through some of the research itself they've done on the web, I don't think there's a system in your body that vitamin D doesn't have some effect on it
  Over 65 years age group

## Views about sunshine and sun exposure

### Sunshine/sun exposure

Respondents held strong views about this complex issue, discussing risks and benefits of sun exposure and, in particular, linking exposure with skin cancer. People reported “conflicting messages” and asked “where's the balance?” and “how do you know when you've got enough [sun]?”. They saw the need for a nuanced public health message and drew distinctions between sunshine and sunbathing—“I think sunshine helps [raise levels in the blood] but I'm not sure you need to sunbathe for it.” A public health message promoting more sun exposure would need to be strong enough to counter the widespread public perception that sun “was terrible for you.” Some felt wider holistic benefits of being outside were being lost because, for example, “a walk across the park makes you feel great!…if the sun is out…you come back feeling more alive and exhilarated!”. Being “out in nature” gave you “the whole package.”

The South Asian and over 65 years age groups had marked differences in attitudes towards sunshine. The latter expressed a particular enthusiasm for sunshine, sharing stories of their youth when their parents told them to stay outside to “get their vitamin” or how they used vitamin D as an excuse to go sunbathing. Conversely, South Asian participants described an aversion, opting to avoid sunlight when possible (box 4).

Box 4 **Sunshine and sun exposure**

Conflicting messages about sun exposure:

- Well it's conflicting messages, cos wherever you are, you're told not to be sunbathing, because it's bad for your skin, because too much sunbathing will, you know, leads to skin cancer and other certain types of cancer as well, so its conflicting messages, because on one hand, if you're saying you have a vitamin D deficiency, you're saying you should get as much sun as possible, but then, where's the balance, of what is, as much sun, you know, how, how do you know you've got enough?
  South Asian group
- I think, while it does some good for you, there are also risks and we're bombarded with a lot more information about skin cancer and staying out of the sun. So if you we're trying to promote vitamin D you'd have to go against that sort of argument that sunlight is terrible for you
  Healthy group
- Interviewer: What about if we were to start saying that everyone should be out in the sun for at least half an hour a day. Do you think that would be something that people would accept as a sensible suggestion?
- Respondents: ‘Delighted’, ‘Yeah’
  Healthy group
- And then I had a blood test that showed I had a deficiency, and it was suggested I took some vitamin D tablets. But I much prefer to have a sunshine (holiday)
  Black British group

South Asian aversion to sun exposure:

- When I'm sitting in the garden, or walking outside, I prefer the shady side
- Shade, walk in the sun (laughs)
- Put a hat on ((other's laughing))
- So in future, I shall try to sit more in the sun, (laughs) which I don't really like, another thing, sitting in the sun gives me a headache, so I'm sitting in the sun, but in the shade if you know what I mean
  Participants from South Asian group

### Government recommendations

No participants were aware of any current government recommendations concerning vitamin D. Many wanted not just recommendations, but justification and evidence to back them up—“detail”—that would allow them to interpret, and more importantly adapt these for their own use. They emphasised the importance of a “clear” message that is “not too generalised”, detailing “why?” and “how long?”. They would welcome guidance—a sort of algorithm—that accounted for vitamin D production, skin cancer risk, skin colour and season, allowing an individual to ‘look up’ a recommended sun exposure tailored to themselves (box 5).

Box 5 **Government recommendations**

- As a consumer of health information I get very upset when I'm given random statements without any theory behind it. Because it means that you as a consumer, have got no idea where to go with that, if you think actually I don't fancy following that advice entirely, you don't quite know how to tweak it for your own ends if you see what I mean. “This is bad, you can't do it” it kind of gives you nowhere to go
  Healthy group
- It's a bit like the weight height chart thing. You could do that with the sun and the months, so you could pinpoint how long you should stay out in what season
  Healthy group
- While it [the sun] does some good for you, there are also risks and we're bombarded with a lot more information about skin cancer and staying out of the sun. So if you we're trying to promote vitamin D you'd have to go against that sort of argument that sunlight is terrible for you
  Healthy group
- As I said before, if you say “moderate” something boosts your vitamin D, then that's fine, but if [a recommendation] is so generalised then it ceases to mean anything. So I think a bit of detail is helpful
  Over 65 years age group

## Attitudes towards testing and supplementation with vitamin D

Many talked about vitamin D in strongly medicalised terms, a perspective that seemed to have arisen from encounters with health professionals. These respondents had a “*test me and treat me*” perspective. One talked about “always having the blood test, regular,…to check the vitamin D level”, something that they had done as a family, “same with my two daughters.” Another wanted the vitamin D test as part of the “annual MOT…with cholesterol and things like that.” Vitamin D was talked of as a medicine or drug, with the respondent wanting “to know what the *side effects* would be if the *dose* became too high”, and to know that taking it “wouldn't include irreversible changes, like kidney failure.” Another wanted to be tested to “know what is the baseline against which any changes would be measured.” Another wanted “a proper diagnosis first.” One comment illustrated how the medical profession had contributed to medicalising vitamin D by drawing an analogy with cholesterol and statin prescription saying “you have your blood test and you know you're short on this or that, and they put you on statins or vitamin D, and that's it, you just take it, at his [the doctor's] word.”

By contrast, others whose attitude might be described as “*only test me if you think I'm ill*” strongly rejected this, drawing comparisons with other vitamins, notably vitamin C,—“If it's a medicine then test, if it’s like vitamin C, no need.” One argued that there was no need to test if you did not feel unwell and looked after yourself—“I wouldn't even think of having my vitamins tested, or anything else tested, unless I was feeling really odd or I was unwell. Because I eat reasonably OK…it wouldn't cross my mind.” (box 6).

Box 6 **Medicalisation of vitamin D**

Medicalised talk about vitamin D:

- I mean, I've always had the blood test regular, and same with my two daughters and they do check the vitamin D level
  >65 years age group
- I'd like to know what the side effects would be if the dose became too high, and that it wouldn't include irreversible changes like kidney failure
  >65 years age group
- I'd want to be tested before I was given a supplement. (murmurs of agreement) I'd want to know what is the base line against which any changes would be measured
  >65 years age group
- Have the test with your cholesterol and things like that, like an annual “MOT”
  Healthy group

Views countering medicalisation:

- If it's a medicine then test, if it’s like vitamin C, no need
  African group
- I wouldn't even think of having my vitamins tested, or anything else tested, unless I was feeling really odd or I was unwell. Because I eat reasonably OK, you know, it wouldn't cross my mind
  Healthy group
- You’re making it a bit different just in the way that we’re discussing it here possibly. Usually we talk about vitamin C, it’s in oranges and it's something very straightforward, and if you know that if you're not eating enough fruit, then maybe have a…or you’ve got a cold, take some extra vitamin C and it might be doing you good. But if you put it in the context of it's a medicine for you to take if you’re sick, then I'd rather know if I needed that medicine first, and then take it
  African group

### Tablets and injections

Vitamin D combined with calcium was the most common formulation (eg, calcichew) reported by respondents. Dislike for these tablets was clear—“they're great big white tablets, four-a-day—like a horse.” “Disgusting,” “slimy,” and “unpleasant” were common descriptions.

Most white and black participants held negative views about injections, which were painful, invasive, and once “pumped into me” offered less “control” and more side effects. However, there were exceptions. Some perceived yearly or three-monthly injection more convenient in the long term. One white woman in the over 65 years age group felt tablets were not “natural”; she preferred injections, especially if she needed long-term supplementation. Respondents in the South Asian group preferred injections. They drew comparisons to the influenza jab and viewed these as a solution to the risk of forgetting daily tablets. There was a general belief injections are more “direct” or “powerful.” Most held negative attitudes towards tablets, believing it is unhealthy to take too many “unnatural” substances, although they did not feel this way about injections.

Many participants expressed a preference for “natural” methods. They would prefer a recommendation of more sunshine and oily fish rather than tablets or injections (box 7).

Box 7 **Attitudes to supplementation**

Injections versus tablets:

- It's like the contraceptive pill vs. the injection. A lot less women have the injection than there is that take the contraceptive pill. I suppose you feel like you've got more control over it if you take something every day
  Healthy group
- Once the damned thing has been pumped into me, I can't do anything about it, but I can stop taking pills. I would be anxious…if I did experience a bad reaction…
  Healthy group
- I'm kind of lazy but compliant (laughter) so the other thing would be how long do you expect me to be deficient in vitamin. If it's going to be for the rest of my life then I'd probably like a yearly injection so I don't need to think about it, carry my pill around, then go on holiday and find I've forgotten them
  Healthy group
- No, I'd rather not have a tablet you take every day because I'd keep forgetting
  >65 years age group

South Asian views on superiority of injections:

- If you're injecting, you're injecting directly in to your veins or your thing and it’s getting to where it needs it better, rather than you taking tablets and then your system has to digest those tablets, so it's that bit longer…so it's more direct
  South Asian group
- Taking of tablets or things, it's not a natural thing that you're [ingesting], it's something that has been generically [sic] produced,…taking too many tablets is not good for oneself anyway
  South Asian group

Preference for “natural” methods to normalise vitamin D status:

- The first thing if…you were deficient, I would initially try to change the diet first, before taking the tablets or any injection. And I think it's only after a period of active sort of dietary intervention would I probably go to [tablets or injections]
  Healthy group
- With me I like to have it as a food. [We need] more education which food contains vitamin D
  South Asian group

### Attitudes to food fortification

Universal food fortification brought out strong views. Most held negative views and regarded the case for fortification as not made. Central issues included freedom of choice—“not forcing it upon people by the Government putting out legislation that all bread has to have vitamin D,” aversion to “unnatural” or tampered foods, lack of trust in the government and food industry—“in the States when you see it on TV, it's coming from the manufacturers”—and fears of overdosing—“large increases in the amount of vitamin D can also be harmful.” Fortification was the government “using a very hard hammer to crack a very small nut.”

This libertarian argument was countered by a significant minority of healthy participants who held a utilitarian view that potential health benefits to vulnerable groups outweighed concerns. Some pointed out this was no different to mass school malt or cod liver oil fortification campaigns of the past. One retired social worker summarised the ‘pro’ fortification side by referring to her own experience—“my Bengali babies really, because there was an identifiable group that was extremely hard…and their mums, extremely hard to get to in educational terms, and who were clearly vulnerable.”

Curiously, the example of fluoridation of water was used by both groups—(for): “it's the fluoride argument again…I understand the reservations, but on balance I'd support it, as I do fluoridation on the basis of the…benefit outweighs the libertarian concern” and (against): “like fluoride in the water, the same argument really…if there's choice, you can have choice.” (box 8).

Box 8 **Attitudes to food fortification**

Negative libertarian attitudes: choice, risk of side effects, government and industry control:

- I think *you should have the choice*, like if you have vitamin D in your bread or without, like if you're going to buy it, *not forcing it upon people* by the Government putting out legislation that all bread has to have vitamin D
  White British group
- It was *like fluoride in the water, the same argument* really…*if there's choice, if you can have choice*
  White British group
- And there is some evidence suggesting that excessively *large increases in the amount of vitamin D can also be harmful*, and I do think that if you were to start such a thing, in the nature of government starting programmes of this kind, (1) you would be using a very hard hammer to crack a very small nut, and applying something to everyone where it might well *not be necessary for everyone*, and where there might be rather *better alternatives* in terms of identifying and educating those who might be at risk (murmurs of agreement)—we could be having a whole battery of measures being applied by government to tell us what to do
  Healthy group
- In the States when you see it on TV and *it's coming from the manufacturers*…it's just these commercial companies…I mean some lobbyist they would have got the ear of government in America, who manufactures the vitamin D which goes into the milk and all that
  >65 years age group

Positive utilitarian attitudes:

- Well, I mean, on the assumption that through normal food consumption you can't OD on it, I think it's the sort of fluoride argument again, isn't it, about medication? You know, I understand the reservations about that, but on balance I'd support it, as I do the fluoriding, the fluoridation. On the basis of the generality of benefit outweighs the, if you like, libertarian concern
  Healthy group
- I wouldn't object to it, because I've read that, for example, in Scotland where there is less sun per year that people are vulnerable to things like rickets. And even dark skinned people, black people, they don't…can't absorb what comes from sunlight as easily because their skins originally made it in a hot climate…
  >65 years age group
- So I think if it could prevent things like rickets and weak bones in old age, I wouldn't have any objection to it
  >65 years age group
- But I go back to sort of my Bengali babies really, because there was an identifiable group that was extremely hard…and their mums, extremely hard to get to in educational terms, and who were clearly vulnerable. The “old stylist” in me really wanted to impose goodness (slight laugh) or impose health…I would support a modest medication or supplementation
  Healthy group

Most favoured education about vitamin D and targeting people through testing over population-wide fortification. Even when one group learned the prevalence of vitamin D insufficiency in the UK, they still believed education or targeted testing should be attempted first (box 9).

Box 9 **Alternative solutions to food fortification**

Health information campaigns:

- Surely the approach should be, publicise those facts [about deficiency] with the notion of the kind of things one can get it, in sunshine, the works, big health programme
  >65 years age group
- Surely it's better to encourage people to have fresh vegetables and fresh food, rather than [fortification] because you don't know what people are eating, so they could easily overdose on it. ‘Yeah, fresh food rather than adding things.’ ‘Fresh fish is the best thing’
  Three participants, >65 years age group

Targeted testing and supplementation:

- Too much vitamin D can have a negative effect, I'm not sure what too much fluoride can do to your teeth, ((brief laugh)) but if too much vitamin D, your body can't process it, I don't think you can go giving that as a blanket, to everybody because then you may end up making some people ill, so it's probably better to test and find out those who need it, then let them have it
  Black British group

Practicalities of implementing food fortification prompted debate on which foods should be fortified and questions about the government's ability to effectively target groups needing vitamin D—“if you're eating Pot Noodles all day, you're not going to head for the [foods with] vitamin D enhancement.” Participants believed consumers should be offered a choice of vitamin D and non-vitamin D brands if mass fortification were implemented; however, if fortified foods were more expensive, they questioned whether these would be bought and hence, would fail to impact on target groups’ consumption of vitamin D.

## Discussion

### Summary of key findings

We found variable but generally limited knowledge of vitamin D, confusion about risks and benefits of sun exposure, a strongly medicalised discourse, and opposing libertarian and utilitarian views on universal food fortification. Perspectives varied little by ethnicity, age or disease group, although South Asians were notable for their dislike of sunshine and preference for injections over tablets.

### Strengths and weaknesses of study

A qualitative approach allowed exploration of attitudes and beliefs about vitamin D. Validity was enhanced through reflexivity (multiple professional perspectives in the research team) and developing the framework of codes through group discussion. Reliability was ensured in several ways. We actively recruited participants from ethnic groups in east London, thus allowing for representation of a broad range of perspectives. Interviews were digitally recorded and professionally transcribed, eliminating potential bias through the note-taking and researcher transcription. Inter-rater reliability was achieved through comparison of individually coded transcripts. MaxQDA software allowed systematic searches through data to retrieve relevant sections.

The main limitations of our work are the relatively small numbers of people from individual ethnic groups, an under-representation of men, and recruitment from a single general practice. Some caution is warranted about the generalisability of findings.

### Comparison with other data

Lack of knowledge regarding vitamin D echoes the findings of two Australian surveys.^20^ ^21^ Limited understanding is unsurprising given the challenges of getting valid information from the internet, limited access to advice from health professionals (who may themselves be ill-informed), ongoing scientific uncertainty about clinical effects of vitamin D, and the lack of public health information campaigns.^22^ Positive attitudes to sun exposure in older people in our study is mirrored by the findings of an Australian study where a large majority of older people believed exposure to sunlight was healthy.^23^ These probably reflect public health messages prevalent in mid to late 20th century.

Confusion—and need for clear advice—about risks and benefits of sun exposure has been explored by the Cancer Society of New Zealand who developed healthy sun exposure messages (SunSmart) accounting for season, skin type and time of day, and Samanek *et al* who found it possible for fair-skinned Australians to produce sufficient vitamin D without unacceptable risks of skin cancer during winter months, although it should be noted that the sun exposure times vary according to latitude and other factors.^24^ ^25^ South Asians described a dislike of sun exposure, a view noted in Chinese women who held negative attitudes towards sunlight despite knowledge of vitamin D production.^26^ South Asian women in New Zealand avoid sunlight due to skin cancer public health messages and a desire to prevent darkening of skin. Half of these participants would spend more time in sunlight if they did not fear skin cancer.^27^

Medicalised talk about vitamin D was prominent in our study, notably from respondents who were positive about being tested and prescribed vitamin D, perspectives which seemed to stem from their interactions with health professionals. These qualitative observations are mirrored by the rapid increase in numbers of people prescribed vitamin D in the area where the study was carried out. The combination of lay interest, medicalisation and clinical uncertainty about the significance of vitamin D status may together be an important influence on upward trends in its testing and prescribing. Medicalisation has not previously been described in relation to vitamin D, but is a well-recognised phenomenon in medicine and society.^28^ Recent clinical trials and meta-analyses showing beneficial effects of supplementation (eg, in children, the elderly, people with COPD)^16^ ^17^ ^29^ are likely to lead to further recommendations for targeted testing and supplementation, which, while potentially appropriate in high-risk groups, may in turn, lead to further medicalisation. Of interest is a recent 26% decline in prescribing in one east London borough (from 133 to 97 patients per 1000 patients from 2011 to 2013) following the introduction of clinical guidance recommending prescribed supplementation only for acute treatment of people with deficiency and over-the-counter supplementation for longer term use (figure 1).

The unpalatability of calcium and vitamin D tablets was striking, adversely affecting adherence.^30^ ^31^ A complex interplay of factors are recognised in compliance with prescribed medications.^32^

We found opposing libertarian and utilitarian views on food fortification. There is accumulating evidence that universal vitamin D food fortification can improve serum 25-hydroxyvitamin D levels,^33–36^with recent studies investigating novel methods of supplementation^37–40^ However, food fortification on a national basis may be unviable if a large proportion of the population disagrees on principle.^41^ ^42^ Arguments were similar to those against fluoridated water,^33^ with freedom of choice, coercion, safety implications and trust issues commonly highlighted.

### Summary

Our findings highlight the need for easily accessible reliable information for the public about vitamin D, and clear, detailed public health messages about sun exposure. Public health messages about sun exposure and vitamin D need to differentiate between the advice for the general population and those at high risk of vitamin D deficiency.^43^ Lay interest, medicalisation and clinical uncertainty may fuel recent increases in testing and prescribing of vitamin D. Plans for food fortification would need to address its unacceptability among a significant portion of the population.
